# The Ministry of Health Bill

**Published:** 1919-03-01

**Authors:** 


### The Hospital

The Workers' Newspaper of Administrative Medicine and Institutional Life, Administration, National Insurance and Health.

No. 1708, Vol. LXV. SATURDAY, MARCH 1, 1919. PRICE TWOPENCE

### THE MINISTRY OF HEALTH BILL.

The Ministry of Health Bill has secured an early appearance in the new Parliament, and there is a general conviction that it is to be pressed forward with all possible speed. The existing industrial position may exercise some prejudice to legislative movement, but, on the other hand, the new rules of procedure will favour progress. Whatever may be true of these rules in other respects, there will be little opposition to the proposal to refer Bills demanding special technical knowledge to committees of experts rather than to leave them to be discussed at large and in detail by the House of Commons. In the past this latter procedure has frequently meant the triumph of the obstructionist and the faddist, and the paralysis of the legislative machine. There is one necessity of the new proposal which requires to be emphasised. It is that, in addition to the experts charged with the discussion in detail of such a Bill as the establishment of a Ministry of Health, the committee shall also include a certain number of members who may fairly be regarded as representing the public interest. We have great respect for the opinions of the experts in their own special departments, and they alone can supply the sound knowledge without which a wise direction cannot be attained. But the expert is not always distinguished by a sense of proportion or by a well-adjusted perspective. Hence the necessity that his proposals shall be reviewed by those who, though not so well equipped with intensive knowledge, are able to cultivate a wider outlook. In the present instance we do not doubt that the committee appointed to consider the Bill will be wisely chosen. Dr. Addison knows the weaknesses as well as the virtues of the profession of which he is a member, and he has now had a considerable experience of legislative and administrative work. He may therefore be trusted to see that the fortunes of his proposals are placed where they will receive adequate discipline, and he is well aware that to secure success they must command both professional and public support. That they will be keenly scrutinised by the medical profession is in the nature of things, and the scrutiny, we are convinced, will not be guided purely by a class interest. The profession as a body is a trustee for the health of the community, and on occasions not a few it has shown itself fully equal to this responsibility.

It is an unhappy circumstance, when so much new legislation is impending, that the medical profession has no generally accepted organisation competent to speak with an authoritative voice on its behalf. The time is marked by the multiplication of new organisations rather than by an endeavour to strengthen those already established. Yet, after all, too much may be made of the story of medical disunion. Here, as in other spheres of activity, small sections are able to produce loud voices, and the platform orator is often of little importance away from his pedestal. The great majority of the profession are busily engaged in the discharge of their important responsibilities, and take little part in the excitements of so-called medical politics. We do not say that such a position is a pure advantage either to the profession or to the public, and, indeed, we think it ought to be modified. But it does mean that in the last resort legislative proposals affecting the profession have to deal with a body of reasonable and common-sense men, who, while perfectly and properly awake to their own interests, ardently desire the triumph of medicine alike in the preventive and the curative direction.

This body of opinion is largely a silent one, and hence politicians have at times believed it to be non-existent, and this to the prejudice of wise administration. We have little sympathy with medical organisations which profess a mere ambition to promote a class interest. But we are convinced that it would be a public gain were the profession able to bring to a united voice the opinions and experience of its largely silent members, for none knows better than these the conditions which exist to-day as hindrances to the physical and moral welfare of the nation. Possibly it would then be revealed that these hindrances are not so much medical as industrial or social or economic, and we are not sure that all the politicians would welcome this demonstration.

When the Ministry of Health Bill becomes an Act it will, in itself, do little more than create machinery, and the application of this will be a matter of administration. The Minister will have powers in the shape of Orders in Council to "take all such steps as may be desirable to secure the effective carrying out and co-ordination of measures conducive to the health of the people." The programme is a wide one and is vague withal. Everything will depend on its interpretation. "Consultative Councils" are to be constituted for the purpose of giving advice and assistance, but here again no precise definition of status and responsibilities is afforded. The Bill, in short, is little more than a sketch plan, and it leaves all structural details to be filled in by the new Minister. We accept it as a start in the right direction, but both the public and the medical profession will have to give alert attention to later developments if the ambition which the Bill professes is to be fully realised.

